# Supplementary material for: Inhibition of Cyclin-Dependent Kinases 8/19 Restricts Bacterial and Virus-Induced Inflammatory Responses in Monocytes
Source: Viruses. 2023 May 31;15(6):1292. doi: 10.3390/v15061292 (PMC10305654; doi:10.3390/v15061292)
Supplement: Supplementary file 1 [file viruses-15-01292-s001.zip › viruses-2362221-supplementary.pdf]

A

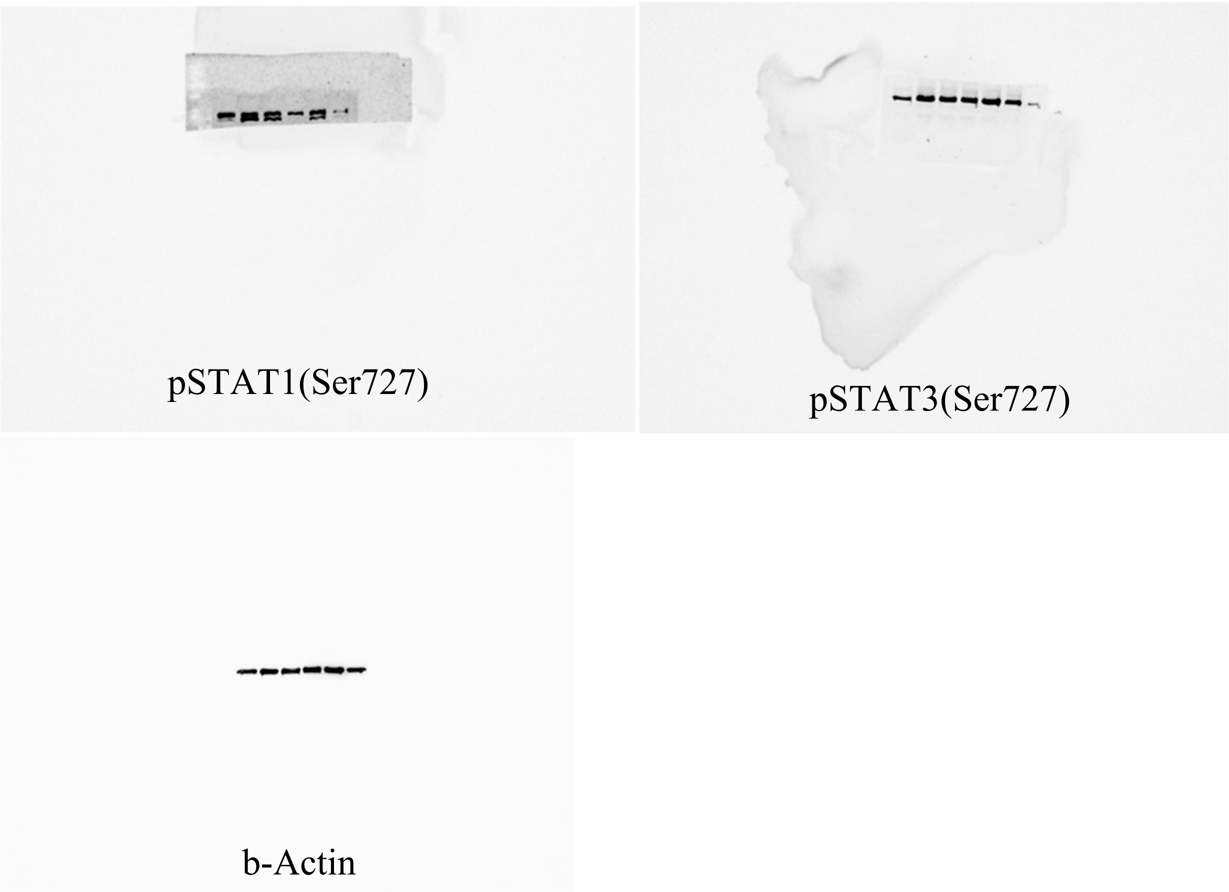

B

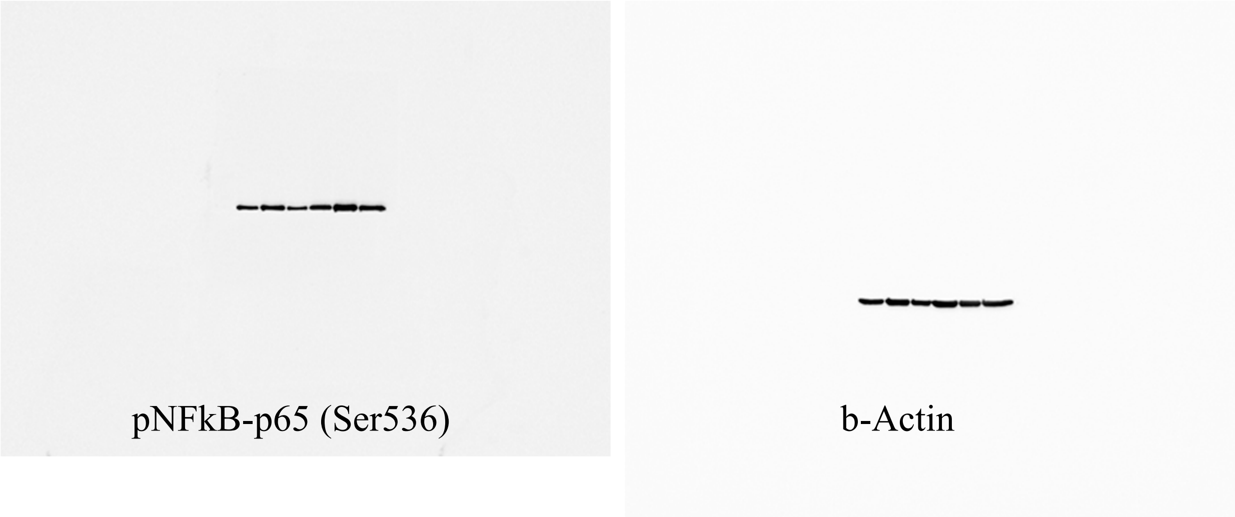

Supplementary Figure S1. Original images for western blot experiments presented on Figure 2. A. Experiment 1: pSTAT (Ser727), pSTAT3 (Ser727), β-Actin. B. Experiment 2: pNFkB-p65 (Se536), β-Actin. The order of the samples (from left to right): Untreated (Control), LPS, Virus, Senexin B, Senexin B + LPS, Virus, Virus + Senexin B
